# Non-steroidal anti-inflammatory drugs for the treatment of pain and immobility-associated osteoarthritis: consensus guidance for primary care

**DOI:** 10.1186/1471-2296-13-23

**Published:** 2012-03-20

**Authors:** Ade Adebajo

**Affiliations:** 1 Academic Rheumatology Group, Faculty of Medicine, University of Sheffield and Barnsley Hospital NHS Foundation Trust, Gawber Road, Barnsley S75 2EP, UK

## Abstract

**Background:**

Osteoarthritis is a common presentation in primary care, and non-selective non-steroidal anti-inflammatory drugs (sometimes also referred to as traditional NSAIDs or tNSAIDs) and selective cyclo-oxygenase 2 inhibitors (COX-2 inhibitors) are commonly used to treat it. The UK's National Institute for Health and Clinical Excellence (NICE) recommends taking patient risk factors into account when selecting a tNSAID or a COX-2 inhibitor, but GPs have lacked practical guidance on assessing patient risk.

**Methods:**

A multi-disciplinary group that included primary care professionals (PCPs) developed an evidence-based consensus statement with an accompanying flowchart that aimed at providing concise and specific guidance on NSAID use in osteoarthritis treatment. An open invitation to meet and discuss the issue was made to relevant healthcare professionals in South Yorkshire. A round table meeting was held that used a modified nominal group technique, aimed at generating opinions and ideas from all stakeholders in the consensus process. A draft developed from this meeting went through successive revisions until a consensus was achieved.

**Results:**

Four statements on the use of tNSAIDs and COX-2 inhibitors (and an attached category of evidence) were agreed: 1) tNSAIDs are effective drugs in relieving pain and immobility associated with osteoarthritis. COX-2 inhibitors are equally effective; 2) tNSAIDs and COX-2 inhibitors vary in their potential gastrointestinal, liver, and cardio-renal toxicity. This risk varies between individual treatments within both groups and is increased with dose and duration of treatment; 3) COX-2 inhibitors are associated with a significantly lower gastrointestinal toxicity compared to tNSAIDs. Co-prescribing of aspirin reduces this advantage; 4) PPIs should always be considered with a tNSAID and with a COX-2 inhibitor in higher GI risk patients. An accompanying flowchart to guide management was also agreed.

**Conclusions:**

Individual patient risk is an important factor in choice of treatment for patients with osteoarthritis and the consensus statement developed offers practical guidance for GPs and others in primary care. Where there are clinical uncertainties, guidance developed and agreed by local clinicians has a role to play in improving patient management.

## Background

Osteoarthritis is a common presentation in primary care, responsible for an estimated 2.4% of all GP consultations in the UK, and a major contributor to the annual 10.1 million consultations for musculoskeletal conditions overall [1]. Those with osteoarthritis have an increased risk of death from any cause, and particular for mortality related to cardiovascular disease and dementia [2].

Traditional non-steroidal anti-inflammatory drugs (tNSAIDs) are effective drugs in relieving pain and inflammation associated with osteoarthritis and other musculoskeletal conditions, and in promoting mobility and physical activity. They are commonly prescribed in primary care. Agents that selectively inhibit cyclo-oxygenase 2 (COX-2 inhibitors) are equally effective [3-6].

In its guidance on osteoarthritis the National Institute for Health and Clinical Excellence (NICE) recommends initial management with education, advice and information, strength and aerobic exercise, and weight loss for overweight and obese patients, followed by treatment with paracetamol or topical NSAIDs if initial treatment is not successful [7].

Where paracetamol or topical NSAIDs are ineffective for pain relief, NICE suggests consideration of an oral non-selective NSAID or a COX-2 inhibitor, prescribed with a proton pump inhibitor (PPI). The NICE guidance suggests taking individual patient risk factors including age into account when selecting a tNSAID or COX-2 inhibitor, with assessment and ongoing monitoring of risk factors.

While the effectiveness of both tNSAIDs and COX-2 inhibitors is similar, the potential adverse effects vary. In particular COX 2 inhibitors are associated with a lower risk of gastrointestinal adverse effects compared to tNSAIDS, and there is some evidence that naproxen is associated with a lower cardiovascular risk than other tNSAIDs [6,8].

The NICE guidance is a useful basis for clinical practice, but in their communications with GPs, for example in referral letters and at educational events, rheumatologists in South Yorkshire identified some uncertainty about its detailed application in the wake of rapidly-evolving new evidence on the risks and benefits of tNSAIDs and COX-2 inhibitors. In particular GPs were unsure about how to assess the risk status of patients who could benefit from a tNSAID or COX-2 inhibitor, and so to identify the most appropriate treatment. Following the high-profile withdrawal of the COX-2 inhibitor rofecoxib in 2004 in the wake of concerns about cardiovascular safety [9], and the subsequent withdrawals of valdecoxib (because of a high rate of serious skin adverse effects and concerns about cardiovascular safety) [10] and lumiracoxib (because of severe hepatic adverse events) [11] some GPs believed that all COX-2 inhibitors had been withdrawn.

To address these uncertainties and in the light of additional clinical evidence, we therefore developed an evidence-based consensus statement, and an accompanying management flowchart to provide more specific guidance for GPs and others working with osteoarthritis patients in primary care. The aim of the consensus process was to develop a practical, evidence-based statement, in line with existing NICE guidance that would help GPs to identify the risk status of patients with osteoarthritis and, where appropriate, to provide the most effective appropriate tNSAID or COX-2 treatment for them.

## Methods

The lead physician for the consensus statement (AOA), issued an open invitation by email to relevant local specialists and primary care physicians. In response to this invitation a group comprising a rheumatologist, a consultant cardiologist, a consultant gastroenterologist, a hospital pharmacist and three primary care physicians with an interest in pain and/or rheumatology attended a round table chaired by the lead physician (a consultant rheumatologist). The key requirement for the project was that the major specialties related to this topic were represented. The meeting used a modified nominal group technique in order to generate opinions and ideas from all the relevant stakeholders who had expertise in primary care, rheumatology, cardiology, gastroenterology, pharmacy and pain relief.

Nominal group technique is a decision making method that typically involves an initial silent generation of ideas by individuals, without discussion or consultation with others, followed by an uncritical sharing of ideas, group discussion and then a ranking or evaluation of ideas [12]. It is regarded as an effective method of dealing with a relatively closed issue, as here.

After the meeting the lead physician developed a draft consensus statement and algorithm that went through several successive drafts until a consensus statement and accompanying flowchart was agreed by at least 90% of the participants. In practice disagreements were minor and the consensus statement was agreed by all participants.

The consensus group rated its recommendations (A strongest, D weakest) based on the quality of the supporting evidence (with the strongest being evidence from a meta-analysis of randomised controlled trials, and the weakest being expert opinion).

The consensus statement and the flowchart has been circulated to GPs and used in training activities.

## Results

The consensus statement emphasised that tNSAIDs and COX-2 inhibitors are effective at treating symptoms of pain and immobility associated with osteoarthritis, but that they vary in their potential for adverse effects, particularly GI, cardiovascular, hepatic and renal. This risk varies between individual treatments and increases with dose and duration of treatment [6].

### Recommendations

Four statements were developed for application in primary care, three supported by category A evidence and one by category C evidence (see Table 1).

**Table 1:** Use of non-steroidal anti-inflammatory drugs

Statement	Category of evidence
NSAIDs are effective drugs in relieving pain and immobility associated with osteoarthritis. COX-2 selective agents are equally effective.	A*

NSAIDs and COX-2 inhibitors vary in their potential gastrointestinal, liver and cardio-renal toxicity. This risk varies between individual treatments within both groups and is increased with dose and duration of treatment	A*

COX-2 selective agents are associated with a significantly lower gastrointestinal toxicity (PUBs and dyspepsia) compared to non-selective NSAIDs. Co-prescribing of aspirin reduces this advantage.	A*

PPI should always be considered with a non-selective NSAID and with a COX-2 agent in higher GI risk patients.	C†

*A-Directly based on evidence from a meta-analysis of randomised controlled trials or from at least one randomised controlled trials

†C Directly based on evidence from non-experimental descriptive studies, such as comparative studies, correlation studies and case control studies or extrapolated from meta-analysis of randomised controlled trials, or extrapolated from at least one randomised controlled trial.

**Figure 1:**
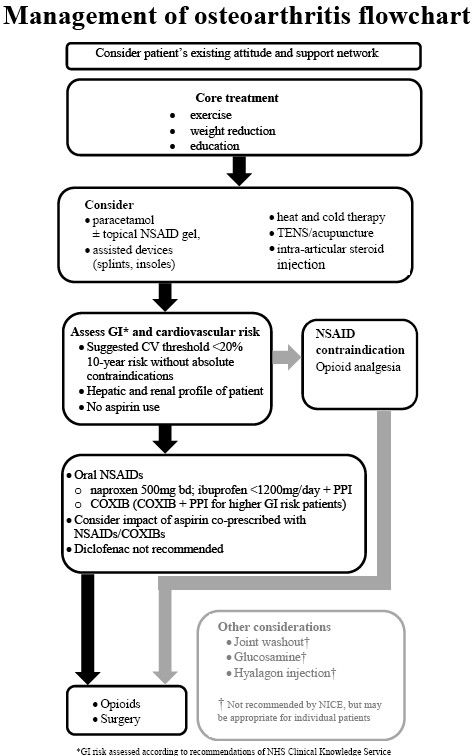
**Management of osteoarthritis flowchart**.

We also developed a flow chart illustrating a clinical pathway for patients with osteoarthritis, based on NICE guidance, and the additional work of the South Yorkshire Clinical Consensus group (see Figure 1).

### Risk prediction

The flowchart recommends calculating cardiovascular risk prediction according to the Joint British Societies guidelines on the prevention of cardiovascular disease in clinical practice (JBS 2), based on calculations derived from the Framingham study [13], and familiar to GPs in other contexts, such as decisions about statin prescribing [14]. They are based on an algorithm that uses a patient's age, systolic blood pressure, total cholesterol to HDL cholesterol ratio, and smoking status to calculate a 10 year risk of cardiovascular disease.

The NHS Clinical Knowledge Service has identified the following patient groups at increased risk for gastrointestinal adverse effects from oral non-selective NSAIDs:

Older age: the risk doubles with every decade after the age of 55

Male sex: the risk of an upper GI complication is twice as high in men than women

History of GI disorder such as gastroduodenal ulcer, GI bleeding

Use of medications such as aspirin, warfarin, oral corticosteroids, selective serotonin reuptake inhibitors, venlafaxine or duloxetine

Serious comorbidity such as cardiovascular disease, hepatic or renal impairment, diabetes or hypertension

Prolonged NSAID use

Use of maximum dose NSAID

Presence of *Helicobacter pylori *infection

Excessive alcohol use

Heavy smoking[15].

The consensus group recommended this guidance as a means of identifying GI risk in patients with osteoarthritis.

The groups identified by the Clinical Knowledge Service are:

Naproxen 500 mg bd or low dose ibuprofen (< 1,200 mg/day) plus a proton pump inhibitor (PPI) are recommended as first choice NSAIDs where patients are at low GI risk and moderate CV risk [7,16,17]. Both ibuprofen and naproxen may inhibit the antiplatelet action of aspirin and so other agents may be preferred in patients already receiving low-dose aspirin for cardiovascular prophylaxis who are likely to be at higher CV risk [18].

### Recent evidence on opioid analgesics

We identified concern about the potential risks of tNSAIDs and COX-2 inhibitors that resulted in some GPs substituting opioid analgesics for osteoarthritis, perhaps unaware of the significant risks associated with opioid use. In the light of new evidence, the consensus statement is cautious on the use of opioid analgesics, and recommends they be restricted to patients with serious or absolute contraindications to tNSAIDs and COX-2 inhibitors [19-21].

Recent research has questioned whether the initial acute efficacy of opioid analgesics is sustained when used for long-term treatment over weeks and months. In addition, since the publication of the NICE guidance in 2008 concern has been expressed about their risk-benefit ratio in long term treatment of chronic musculoskeletal pain. A recent review of more than 36,000 prescriptions found an significantly increased cumulative risk over 12 months of cardiovascular events (myocardial infarction, stroke, hospitalisation for heart failure, coronary vascularisation and out of hospital cardiac death) for patients taking opioid analgesics compared to non-selective NSAIDs (*p *< 0.001) and to COX-2 inhibitors (*p *= 0.004) [21]. There was, similarly an increased risk of fractures, admission to hospital for safety events, and all-cause mortality for those taking opioids compared to non-selective NSAIDs or COX-2 inhibitors. There was an increased risk of upper or lower GI bleeding for opioids compared to COX-2 inhibitors (*p *= 0.04). The number needed to harm reported in this study was small for opioids, and clinically relevant.

### Diclofenac

In a departure from the NICE guidance, which does not differentiate explicitly between different tNSAIDs, the consensus statement explicitly recommends against the use of diclofenac. The decision for this additional recommendation was based on the strength of emerging evidence (largely published after the development of the NICE guidance) suggesting a higher cardiovascular risk such as stroke, cardiovascular death and myocardial infarction with diclofenac than other tNSAIDs and selective COX-2 inhibitors [8,22,23]. This emerging evidence suggests that it is prudent to take a precautionary approach and recommend the choice of one of the several alternative treatments to diclofenac when appropriate for new patients.

A retrospective population-based nested case-control analysis of data from the clinical records of more than 7 million patients registered with 468 UK general practices found a 55% increased risk of MI for those taking diclofenac, compared to those taking no tNSAIDs or COX-2 inhibitors in the previous 3 years (*p *< 0.05) [22]. The increased risk for ibuprofen was 24% and for the now withdrawn selective COX-2 inhibitor rofecoxib was 32% (both *p *< 0.05) [22]. For diclofenac the number needed to harm over a year was 521 treated patients for every additional myocardial infarction, compared to 1,005 for ibuprofen and 695 for rofecoxib. An observational study found a 5.54-fold increase in the risk of death and a 2.24-fold increase in the risk of admission to hospital with myocardial infarction in heart failure patients taking > 100 mg a day of diclofenac [23]. In a recent study of a population of patients who had already had a myocardial infarction, diclofenac was identified as the tNSAID with the highest risk of death or recurrent MI (HR3.26; 95%CI2.57-3.86) - about twice the risk of treatment with any tNSAID (HR1.45;95%CI1.29-1.62) [24].

### Selective COX-2 inhibitors

COX-2 inhibitors were recommended for patients identified to be at risk from GI toxicity but not at significant CV risk (< 20% 10-year risk of an event according to the Joint British Societies risk score [14]). There is evidence that both COX-2 inhibition and use of a non-selective NSAID plus PPI can reduce the risk of upper GI adverse events [25-27], and evidence from a large prospective randomised controlled trial of high risk patients that COX-2 inhibitors may prevent gastrointestinal adverse effects to a greater extent than a combination of tNSAID and PPI [28]. This RCT, of patients with osteoarthritis or rheumatoid arthritis who had a previous gastroduodenal ulcer and allocated to treatment with celecoxib or diclofenac and omeprazole, found a significant difference between the proportion of patients on celecoxib who developed a clinically significant upper or lower GI event (20/2238, 0.9%), and those who developed an event on tNSAID plus PPI treatment (81/2246, 3.8%), *p *< 0.0001 [28].

### Future research

One outcome of reviewing national guidelines in a local context as we did is coming across gaps in recommendations, often because of a lack of supporting clinical evidence. A number of clinical uncertainties were identified in developing the consensus statement, where future research may be warranted. These included:

○ The efficacy, safety and cost effectiveness of COX-2 inhibitors with and without PPI treatment versus naproxen or ibuprofen with and without PPI treatment

○ The CV safety of COX-2 inhibitors versus tNSAIDs, including use of the 20% risk over 10 years threshold for CV appropriate NSAID prescribing.

○ The clinical effects of COX-1 inhibition and the pathogenesis of small bowel damage.

The first of these questions is addressed by the Prospective Randomized Evaluation of Celecoxib Integrated Safety vs. Ibuprofen or Naproxen (PRECISION). It is a large-scale trial expected to recruit 20,000 participants that should provide useful information about cardiovascular safety of non-selective NSAIDs and selective COX-2 inhibitors [29]. Results are scheduled for publication in 2014 [30].

## Discussion

We aimed to produce clear, simple short guidance that was easy to use and developed in a way that meant that local clinicians would have a sense of local ownership. These characteristics have been identified by GPs as factors likely to make them refer to a guideline [31].

Clinical guidelines can help improve the quality of care when there is a lack of clarity about appropriate clinical practice and when scientific evidence can provide an answer [32]. The uncertainty about the use of non-selective, traditional NSAIDs and COX-2 inhibitors recognised by specialists in South Yorkshire makes the development of these guidelines an appropriate response to potential suboptimal management of patients with osteoarthritis. The consensus statement and accompanying flowchart make a number of practical and achievable recommendations to improve clinical treatment in primary care.

When choosing to manage osteoarthritis with tNSAIDS and COX-2 inhibitors, GPs should make an appropriate assessment including ongoing monitoring of GI and CV risk to maximise the benefits and minimise the risks of treatment. This consensus statement identifies practical ways for GPs to do that. Risk profile can be influenced by a number of factors including dose, concurrent aspirin use and age [33]. The lowest effective dose should be used for the shortest possible duration, as chronic treatment is associated with increased risk [34].

The reasons for the successful development of this local consensus statement include its restricted focus on a particular area of clinical uncertainty, its involvement of a wide range of stakeholders, and the delivery of a concise and practical clinical pathway.

## Conclusions

National clinical guidelines are effective ways to bring about improvements in the quality of care, but they do not always provide the practical guidance that primary care professionals require. Where there are uncertainties in clinical practice that might affect the quality of patient care, local guidance developed by local groups of clinicians has a role to play in health improvement.

Ascertaining individual patient risk is important when choosing treatment for patients with osteoarthritis, and the South Yorkshire consensus statement provides practical guidance for GPs and others in primary care to measure risk and guide therapeutic decisions.

## Competing interests

AA has received unrestricted educational funding from various pharmaceutical companies including Pfizer. Funding for the medical writing of this manuscript was provided by Pfizer.

## Authors' contributions

AA had involvement in all aspects and stages of the drafting, completion and approval of this manuscript.

## Pre-publication history

The pre-publication history for this paper can be accessed here:

http://www.biomedcentral.com/1471-2296/13/23/prepub
